# Differential Signatures of Second Language Syntactic Performance and Age on the Structural Properties of the Left Dorsal Pathway

**DOI:** 10.3389/fpsyg.2017.00829

**Published:** 2017-05-23

**Authors:** Kayako Yamamoto, Kuniyoshi L. Sakai

**Affiliations:** ^1^Department of Basic Science, Graduate School of Arts and Sciences, The University of TokyoTokyo, Japan; ^2^Japan Society for the Promotion of ScienceSaitama, Japan; ^3^Japan Agency for Medical Research and Development – Core Research for Evolutional Science and TechnologyTokyo, Japan

**Keywords:** diffusion MRI, white matter, dorsal and ventral pathways, language acquisition, syntax

## Abstract

In adult second language (L2) acquisition, individual differences are considerably large even among people with similar experiences. The neural mechanisms underlying this variability would include structural plasticity of language-related pathways. To elucidate such neuroplasticity, we focused on the transitional period of adolescence, which is associated with certain plasticity toward maturation following the sensitive period of language acquisition (≤12 years old). The adolescent brain would thus be influenced by age-dependent factors, as well as performances in L2. Here, we examined individual differences in L2 performances controlling the duration of experience to reveal the differential signatures of performances and age on the plasticity of structural properties in major language-related pathways. We recruited Japanese students at two ages, i.e., junior (age: 13–14) and senior (age: 16–17) high-school students, all of whom started to expose to English at age 12 or 13. We divided them into subgroups, so that either L2 performance [Junior (High)/Senior (Low)] or age [Senior (Low)/Senior (High)] was matched in group comparisons; the duration of L2 experience was also controlled between the Senior (Low) and Senior (High) groups. We then examined the thickness and fractional anisotropy (FA) of the dorsal and ventral pathways, i.e., the arcuate fasciculus (Arcuate) and inferior fronto-occipital fasciculus (IFOF), respectively, using semi-automatic methods for selecting regions without branches. Regarding FA in the left Arcuate, the Senior (High) group showed significantly higher FA than the other two groups, indicating performance-related group differences. Further, FA in the left Arcuate was selectively correlated with the accuracy of a syntactic task. Regarding the thickness of the left Arcuate, the Senior (High) and Senior (Low) groups showed significantly larger thickness than the Junior (High) group, indicating age-related group differences. These differential performance-related and age-related signatures were evident on the left Arcuate alone, in contrast to the right Arcuate that showed only mild differences in thickness, and to the bilateral IFOF that lacked either signature. Our results suggest that the left dorsal pathway continued to develop to adolescence, and that performance differences in a syntactic task can be predicted by its FA, independent of age and the duration of experience.

## Introduction

Second language (L2) acquisition shows considerably large individual differences, especially when the L2 is acquired in adulthood. Even among people with similar L2 experiences (e.g., taking the same classes or lessons on a foreign language), some improve their L2 performances in a relatively short period of time, while others do not improve as well. This is in marked contrast to first language (L1) acquisition, in which linguistic abilities are similar among individuals despite highly variable experiences. However, individual differences in L1 do emerge when effortful language use is imposed. By using verb-, rhyme-, and opposite word generation tasks in L1 for 9-year-old children and adult participants, previous studies have reported that distinct regions in the left frontal and occipital cortices showed age-related or performance-related activations (Schlaggar et al., 2002; Brown et al., 2005). These studies have indicated the importance of employing subgroups, in which either age or performance was matched, thereby fixing one of the two factors in group comparisons. Individual differences would also be revealed when people learn to read or write even with their own languages through educational training of specific skills. A previous study has reported that the structural property of a white matter pathway connecting the left temporal and parietal language areas may have plasticity associated with literacy experience even for adults, by comparing three groups of illiterates, ex-illiterates who learned to read during adulthood, and literates (Thiebaut de Schotten et al., 2014). Long and intensive experiences are required also in L2 acquisition, which may be supported by the structural plasticity of language-related pathways. To elucidate such neuroplasticity, it is of interest to focus on the transitional period of adolescence, which occurs after the sensitive period of language acquisition (≤12 years old). Indeed, adolescence has been suggested to involve certain plasticity toward maturation (Fuhrmann et al., 2015). The total cerebral volume has been reported to show a gentle inverted-U trend, with a peak age different among genders (girls: 10.5 years, boys: 14.5 years) (Lenroot et al., 2007). While the gray matter volume gradually decreases in adolescence, after peaking at later years in higher order association areas (Gogtay et al., 2004; Lenroot et al., 2007), the white matter continues to increase until the twenties or later with some regional differences (Westlye et al., 2010). Moreover, developments in white-matter pathways are accompanied by large individual differences. A previous longitudinal study showed that some show increases in volume, while others show decreases, even at similar ages (Lebel and Beaulieu, 2011). The white matter in the adolescent brain would be influenced by multiple factors depending on the participants’ ages (e.g., biological maturation), as well as on the attainment of cognitive/motor abilities. More specifically, in major language-related pathways, the independent factors of age and performances after intensive L2 experiences may be reflected in the plasticity of different structural properties.

Given the large individual differences in L2, many complicated issues should be tackled in examining the neural plasticity related to L2 acquisition. In addition to participants’ current age, the age of first exposure (AOE) and the duration of exposure (DOE) represent other factors in L2 acquisition (Li et al., 2014). In the present study, we controlled AOE in order to examine any group differences related to the current ages of participants (hereafter, age-related group differences), and therefore recruited students at two ages, i.e., junior (age: 13–14) and senior (age: 16–17) high-school students. Their AOE to English was 12 or 13, and they attended the same school where English classes were based on the curriculum guidelines determined by MEXT (Ministry of Education, Culture, Sports, Science and Technology). The temporal factor of L2 experience, as represented by the DOE, was also controlled and eliminated from the performances for the students at the same age. Even among such students, large individual variations in L2 performance were observable. Given that Japanese students tend to make similar mistakes in English, such as applying the null-subject (pro-drop) allowed in Japanese, we examined participants’ syntactic abilities in English by a syntactic error-detection task (Syn) that we previously developed with high school teachers (Sakai et al., 2009). Orthographic knowledge in English was also examined by using a spelling error-detection task (Spe) with basically the same sets of sentences. Based on the task performances, the junior and senior students were separately divided into subgroups. As a result, the task performances of the Junior (High) and the Senior (Low) groups matched, while those of the Senior (High) group were significantly better. We then compared these three groups, with either age or L2 performance being fixed. After the group division, we identified the dorsal and ventral language-related pathways, two major routes that combine multiple language areas (Hickok and Poeppel, 2007; Friederici, 2011). The dorsal and ventral pathways correspond to the fronto-temporal segment of the arcuate fasciculus (Arcuate) and the inferior fronto-occipital fasciculus (IFOF), respectively. We identified these pathways using our previously established semi-automatic methods of defining seeds for tractography in a diffusion magnetic resonance imaging (diffusion MRI) analysis (Yamamoto and Sakai, 2016). We focused on two distinct structural properties, i.e., the thickness (or the volume of a tract) and fractional anisotropy (FA—the degree of myelination and/or fiber organization), to examine how age and L2 performance are reflected in these properties of the dorsal and ventral pathways in each hemisphere.

Several diffusion MRI studies have suggested that the dorsal pathways mature later than the ventral pathways in both infants and children. A diffusion MRI study with tractography has reported that one of the two dorsal pathways, the one connecting the inferior frontal gyrus (IFG) and the temporal cortex, was not trackable in 2-day-old newborns; in contrast, the IFOF of the ventral pathways, as well as the other dorsal pathway connecting the premotor cortex and the temporal cortex, was already present (Perani et al., 2011). In a study with 7-year-old children, both the dorsal and ventral pathways were trackable, but FA of these pathways was significantly lower than that of adults (Brauer et al., 2013), suggesting that the language-related pathways were not fully mature at this age. Regarding the adolescent ages, it has been suggested that the dorsal pathway was still under development, and that the ventral pathway showed less prominent development (Lebel and Beaulieu, 2011). FA in the dorsal pathway has been reported to increase during adolescence, mainly associated with increases in parallel diffusivity, which may reflect the increases in axon diameters (Giorgio et al., 2010). FA in the language-related pathways may increase with linguistic experiences, as previous longitudinal studies reported that FA of the left ventral pathway of bilinguals was higher than that of monolinguals, possibly reflecting differences in semantic aspects (Mohades et al., 2015). However, it remains less clear how these structural developments can be correlated with behavioral measurements. The relatively slow development of the dorsal pathway suggests the interesting possibility that this pathway may be more strongly influenced by language experiences. As suggested by a native language neural commitment theory (Kuhl, 2004), neural networks develop under the coding of L1 inputs from early ages, further influencing the acquisition of L2 in later ages. Indeed, L2 abilities have been reported to be correlated with L1 abilities, for instance in reading proficiency (van Gelderen et al., 2007). Given that improvements, as well as individual differences, in L1 during adolescence become smaller than those in L2, examining the relationships between L2 acquisition and the structural properties would be of worth in understanding the neural plasticity of language-related pathways in adolescence.

We have performed the following functional and structural studies on adolescent students. In our previous functional magnetic resonance imaging (fMRI) study, we identified cortical regions involved in syntactic processing using the same English tasks as in the present study, and showed a positive correlation between the individual activations of the left IFG and the performance accuracy of the Syn task (Sakai et al., 2009). Our voxel-based morphometry (VBM) study further clarified that individual leftward lateralization of a single region in the IFG also showed a correlation with the accuracy of Syn (Nauchi and Sakai, 2009). Moreover, in our recent diffusion MRI study we reported that individual FA in the left Arcuate was correlated with the accuracy of Syn (Yamamoto and Sakai, 2016), suggesting the importance of the left dorsal network including the IFG and the Arcuate in syntactic processing. More specifically, among the Arcuate and IFOF in both hemispheres, we revealed that FA in the left Arcuate alone was positively correlated with the performance, i.e., accuracy of the Syn task, but not with that of Spe or with L1 verbal fluency. Further, within monozygotic twin pairs, neither the accuracy of Syn nor FA in the left Arcuate were significantly correlated between the twins, in spite of the high inter-twin correlation for the thickness of the left Arcuate. Given these results, syntactic abilities in L2 and FA in the left Arcuate may thus have been sensitive to non-shared environmental factors by which the twins were individually affected, while the thickness was dependent on shared genetic/environmental factors. Based on these points, we made two further hypotheses. First, FA in the left Arcuate should show performance-related group differences that would be observable even among the students with the same DOE, because larger variances within the monozygotic twin pairs were observed for FA than for the thickness. Second, the thickness of the left Arcuate should show age-related group differences, as a similar thickness was observed for monozygotic twin pairs. We tested these hypotheses using semi-automatic methods, which we developed previously for defining seeds in tractography and selecting regions of interest (ROIs) (Yamamoto and Sakai, 2016). In this previous study, we reported that the thickness of the left Arcuate, averaged in a one-dimensional ROI, was clearly larger than that of the right Arcuate; such laterality was evident neither for the thickness of the IFOF, nor for FA in the Arcuate or IFOF. In the present study, recruiting a larger number of adolescent participants, we confirmed that the leftward laterality of the Arcuate was consistent among the three groups. We further examined the correlation with individual accuracy of Syn/Spe to clarify which particular aspects of L2 abilities were related to the structural properties that showed any performance-related group differences. Our results should thus help to elucidate the neuroanatomical basis of language acquisition after the sensitive period.

## Materials and Methods

### Participants

Junior high-school students (age: 13–14) and senior high-school students (age: 16-17) were recruited from the Secondary School attached to the Faculty of Education, the University of Tokyo. Twin pairs were included among the participants; this school has performed educational as well as twin research. The following accumulative inclusion criteria were employed in the present study: (i) right-handedness, i.e., a positive laterality quotient (LQ), which was assessed using the Edinburgh inventory (Oldfield, 1971), (ii) with neither hearing/visional problems nor history of neurological/psychiatric diseases, (iii) native Japanese speakers who started to acquire English in formal education at the age of 12 or 13 (this condition was met by most of the students in this school), and (iv) reaction times (RTs) for Spe within the presentation time (6400 ms) for more than 90% of the trials including incorrect responses. To avoid including monozygotic/dizygotic twin pairs with potentially similar characteristics, the one with the higher score in Spe was analyzed for each pair who met these criteria. As regards the first criterion, 8 junior and 11 senior students, who showed a negative LQ or reported a potential history of left-handedness, were dropped. As a result, the population showed relatively strong right-handedness (LQ > 35). One participant each for the second and fourth criterion, and two participants for the third criterion, were dropped.

For the senior high-school students, we employed three additional criteria: (v) an accuracy of Spe higher than 65% (i.e., “mean – 1.5 SD”), (vi) no worse outliers in each task (i.e., higher than the “mean – 2 SD” for the accuracy, and shorter than the “mean + 2 SD” for the RTs), and (vii) shorter RTs for easier Spe than for Syn. Given that most Japanese students learn an alphabetic writing system at the age of 12–13, and that the senior high-school students had been studying English for about 4 years, the fifth criterion was necessary to exclude the potential effects of poor reading abilities and precisely assess individual syntactic abilities; five senior high-school students were dropped for this reason. Two students were dropped because they did not meet the sixth criterion, and two more students because they did not meet the seventh criterion. As a result, we enrolled a total of 39 junior and 38 senior high-school students.

We divided the junior high-school students into two groups: a group of 14 students [the Junior (High) group] who scored higher than 65% in Spe (this was identical to the fifth criterion employed for senior high-school students), and a group of 25 students [the Junior (Low) group] with scores lower than 65% in Spe, which were too low to assess their L2 abilities and related structures further. In regard to the senior high-school students, we first divided the 38 students into two groups using K-means cluster analysis (R software)^1^ on the accuracy of Syn and Spe tasks. This analysis yielded a group of 15 participants with higher L2 abilities [the Senior (High) group], as well as a group of 23 participants with lower L2 abilities. Because the performances of the group with lower L2 abilities did not match those of the Junior (High) group for the accuracy and RTs of Spe (one-sided *t*-tests, *P* < 0.05), we further performed a hierarchical cluster analysis using Ward’s method in the R software^1^ based on the accuracy and RTs of Spe for the 23 students with lower L2 abilities. As a result, we divided these students into two groups: a group of 5 students with the higher accuracy and shorter RTs of Spe [the Senior (Middle) group], and a group of 18 other students [the Senior (Low) group]. The accuracy/RTs of both tasks for the Senior (Low) group matched those for the Junior (High) group (one-sided *t*-tests, *P* > 0.05). Because our purpose was to examine the differences between the performance-matched groups with different ages, as well as between age-matched groups with different L2 abilities, we focused on the Junior (High), Senior (Low), and Senior (High) groups, dropping the other groups. All participants in the Senior (High) group, as well as seven participants in the Senior (Low) group, were included in our previous study (Yamamoto and Sakai, 2016), in which we analyzed participants with an accuracy of Spe ≥ 80% (Experiment I); moreover, these former participants were the subset of the same 38 senior high-school students mentioned above. Participants in the Junior (High) group were newly recruited for the present study. All participants in the three groups were scanned with the same diffusion MRI protocol. Demographic details of participants in these three groups, such as age, AOE, DOE, and LQ, are shown in **Table 1**. We obtained written informed consent for this research from all the students and their guardians. This study was approved by the Institutional review board of the University of Tokyo (Komaba) and by the Secondary School.

**Table 1:** Demographic details of the participants in each group.

		*N*	Age	AOE	DOE	LQ	vf
Junior (High)	Female	7	14 ± 0.5	12.7 ± 0.3	1.5 ± 0.3	82 ± 20	24 ± 3.7
	Male	7	14 ± 0.3	12.6 ± 0.3	1.4 ± 0.2	86 ± 13	20 ± 2.6
	Total	14	14 ± 0.4	12.6 ± 0.3	1.5 ± 0.3	84 ± 16	22 ± 3.6
Senior (Low)	Female	10	17 ± 0.3	12.6 ± 0.3	4.4 ± 0.2	82 ± 21	24 ± 5.6
	Male	8	17 ± 0.3	12.7 ± 0.4	4.4 ± 0.2	92 ± 16	21 ± 5.0
	Total	18	17 ± 0.3	12.7 ± 0.3	4.4 ± 0.2	86 ± 19	23 ± 5.4
Senior (High)	Female	9	17 ± 0.4	12.5 ± 0.4	4.5 ± 0.2	82 ± 16	26 ± 6.5
	Male	6	17 ± 0.3	12.6 ± 0.3	4.5 ± 0.2	83 ± 16	29 ± 5.3
	Total	15	17 ± 0.4	12.6 ± 0.3	4.5 ± 0.2	83 ± 15	27 ± 6.0


*Data are shown as the means ± SD for the number of participants (N), age, age of first exposure (AOE) to English, duration of exposure (DOE) to English, laterality quotient (LQ), and the number of words produced in the verbal fluency task (vf)*.

### Stimuli and Tasks

All the students performed English syntactic (Syn) and spelling (Spe) error-detection tasks (50 trials each for Syn and Spe tasks), as well as a verbal fluency task in Japanese (for three initial letters in hiragana). The tasks were the same as those we employed in our previous studies (Nauchi and Sakai, 2009; Sakai et al., 2009; Yamamoto and Sakai, 2016); the stimuli for Syn are listed in Sakai et al. (2009). In the Syn task, we tested argument structures and related syntactic knowledge, which are difficult for L2 learners to acquire. For instance, as objects of transitive verbs are freely omitted in Japanese as well as other languages, Japanese students tend to accept incorrect (^∗^) English sentences, such as “*Do you often meet Mary? – ^∗^Yes, I often meet*” [see Sakai et al. (2009) for further information on other types of syntactic errors]. Because ungrammatical sentences in the Syn task cannot be judged as incorrect by semantic information alone (e.g., by internally translating English sentences into Japanese), the accuracy of the Syn task reflected individual syntactic abilities appropriately.

### Magnetic Resonance Image Acquisition and Data Analyses

Magnetic resonance images were acquired with the same protocols and parameters as in our previous study (Yamamoto and Sakai, 2016). With the same semi-automatic procedures described in this previous study, we identified the Arcuate and IFOF in each individual hemisphere. In the present study with group comparisons, all the tracts were normalized and analyzed after the tracking in individual brains; in our previous study, tracts were analyzed in the native space to focus on individual variabilities of structural indices (Yamamoto and Sakai, 2016). Using the affine and non-linear transformation with FLIRT and FNIRT, all the tracts were spatially normalized to the Montreal Neurological Institute (MNI) space, and were binarized with the fslmaths function of FSL software. While FLIRT and FNIRT are conventional volume-based registration algorithms using voxel-level information, a method of combining volume-based and surface-based registration (Zöllei et al., 2010), as well as an improved voxel-based registration (Schwarz et al., 2014), has been proposed for better alignment. We overlaid the binarized tracts across the participants for each pathway, thereby producing population probability maps, in which voxel values represented the number of participants. The population probability maps with thresholding (at least half of the participants) were smoothed and shown using MRIcroN software^2^.

### ROI Selection

We basically followed the ROI selection procedures in our previous study (Yamamoto and Sakai, 2016); we determined one-dimensional ROIs (in an antero-posterior direction) at the portion with the most uniform *thickness*, and showed that the appropriate length, i.e., *ROI size*, minimizing the individual variances of thickness was 20 and 15 mm for the Arcuate and IFOF, respectively. We defined the thickness of a pathway as the number of voxels at a coronal section (voxel size, 1 mm^2^), because the relatively straight portions of these two pathways (i.e., the portions without branches) were nearly horizontal. We first measured the thickness of each participant’s Arcuate for the length of 35 mm (*y* = -40 ∼-6; the candidate region for ROI), excluding the branching or curved portions. Within these candidate regions, we then calculated the standard deviation (SD) of the thickness across a tract segment of 20 mm, and slid the segment. At each position, the *averaged SD* was obtained among all the participants of the three groups. We selected the segment with a minimal averaged SD as an ROI in each hemisphere, thereby extracting the region with the most uniform thickness across participants. Using these procedures, we objectively selected ROIs at the same position in the MNI space, thereby minimizing individual variabilities in the ROI selection.

In regard to the IFOF, we set a candidate region with a length of 70 mm and selected a 15-mm-long ROI in each hemisphere, in accordance with our previous study. We measured the thickness of each participant’s IFOF for *y* = -75 ∼-6, excluding the branching or curved portions. Within these candidate regions, we calculated the SD of the thickness across a tract segment of 15 mm, and slid the segment. At each position, the averaged SD was obtained among all the participants. We selected the segment with a minimal averaged SD as an ROI in each hemisphere.

We next examined the correlation with individual accuracy of Syn/Spe to clarify which aspects of L2 abilities were related to the structural property that showed any performance-related group differences. For the analyses within a single group, we aimed to examine individual variances in a pathway, thereby employing individually selected ROIs on the normalized tracts. We selected ROIs where the thickness was most uniform for the tract of each participant in the MNI space. As described above, the thickness of the Arcuate was measured for the candidate region with a length of 35 mm (*y* = -40 ∼-6). Within these candidate regions, we calculated the SD of the thickness across a tract segment of 20 mm, and slid the segment. We selected the segment with a minimal SD as an ROI.

In order to avoid the potential uncertainty of FA near the peripheral regions of fiber tracts, we set the thresholding (FA ≥ 0.2) in accordance with previous literature. The resultant FA maps were normalized using the affine and non-linear transformation with FLIRT and FNIRT. Within each ROI, we calculated the mean FA for each group, and examined the group differences. Statistical analyses were performed using R software. We used the packages cocor^3^ for comparing correlations, ppcor^4^ for partial correlations, and pwr^5^ for power analyses, as well as lme4^6^ and car^7^ for linear mixed-effects models (Koerner and Zhang, 2017).

## Results

### Behavioral Data

**FIGURE 1:**
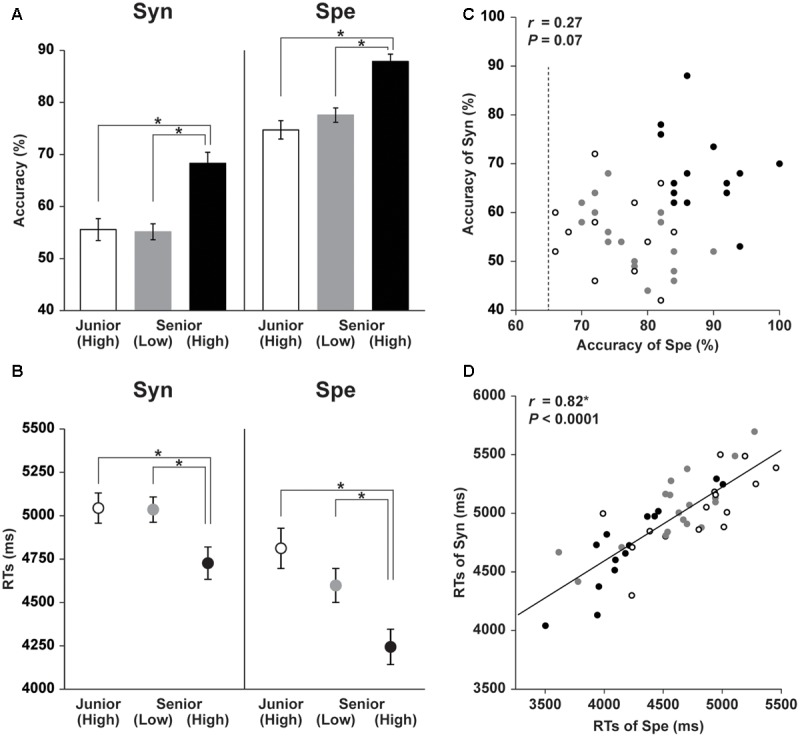
**Behavioral data of Syn and Spe tasks for the three groups. (A)** The accuracy of Syn and Spe tasks for the three groups of high school students, Junior (High), Senior (Low), and Senior (High), are indicated by bars with white, gray, and black shades, respectively. Error bars indicate the standard error of the mean (SEM) for the participants, and asterisks denote the significant differences (Bonferroni-corrected *P* < 0.05). **(B)** The reaction times (RTs) of the Syn and Spe tasks for the three groups are indicated by dots corresponding to the white, gray, and black shades in **(A)**. The Senior (High) group showed significantly higher L2 abilities, i.e., higher accuracy and shorter RTs, while the Junior (High) and Senior (Low) groups showed no significant difference in the accuracy or RTs in either task. **(C,D)** Independence of the accuracy of the Syn and Spe tasks. Individual behavioral parameters were plotted to compare the two tasks, as shown by the plotted dots, whose groups correspond to the shades in **(A)**. The dotted line indicates an inclusion criterion of Spe. The accuracy of Syn and Spe showed no significant correlation **(C)**, while the RTs of Syn and Spe were highly correlated **(D)**.

Task performances of Syn and Spe in the Junior (High), Senior (Low), and Senior (High) groups are shown in **Figure 1**. Regarding the accuracy (**Figure 1A**), a two-way repeated measures analysis of variance (rANOVA) [group × task (Syn, Spe)] showed significant main effects of group [*F*(2,44) = 47, *P* < 0.0001] and task [*F*(1,44) = 169, *P* < 0.0001] without interaction [*F*(2,44) = 0.4, *P* = 0.6]. A linear mixed-effects model analysis [fixed effects: group, task; random effects: subject] confirmed significant effects of group [χ^2^(2) = 68, *P* < 0.0001] and task [χ^2^(1) = 219, *P* < 0.0001]. Regarding RTs (**Figure 1B**), an rANOVA showed significant main effects of group [*F*(2,44) = 6.3, *P* < 0.005] and task [*F*(1,44) = 116, *P* < 0.0001] with a significant interaction [*F*(2,44) = 4.2, *P* < 0.05]. A linear mixed-effects model analysis confirmed significant effects of group [χ^2^(2) = 13, *P* < 0.005] and task [χ^2^(1) = 102, *P* < 0.0001]. One-way ANOVAs did not show a significant effect of gender for the accuracy or RTs of either task [*F*(1,45) < 0.2, *P* > 0.7]. According to the *t*-tests (Bonferroni-corrected for three comparison pairs, significance level at α = 0.017), we confirmed that the Senior (High) group had significantly higher accuracy and shorter RTs than the other two groups in both tasks (effect size: *d* > 0.90, statistical power: 1 – β > 0.73), indicating that the Senior (High) group had better syntactic abilities and word knowledge than the other two groups.

If a behavioral parameter reflected factors commonly involved in both Syn and Spe, such as reading proficiency and task difficulty, that parameter would be correlated between Syn and Spe among the participants (see Yamamoto and Sakai, 2016). For instance, RTs of a slow reader would be relatively long among the participants for both tasks. Conversely, if a behavioral parameter was not correlated between the two tasks, that parameter would reflect, at very least, factors distinctly required in each task. Thus we analyzed the correlations among accuracy of the two tasks, as well as among RTs. The accuracy of Syn and Spe was not significantly correlated (*r* = 0.27, *P* = 0.07; **Figure 1C**); it showed a week correlation among the three groups, as the Senior (High) group showed the higher accuracy of Syn/Spe than the other two groups, but when examined in each group, such significant positive correlation between the accuracy of Syn and Spe was not observed in any of the three groups (*P* > 0.3). These results suggest that the accuracy of Syn and Spe mainly reflected abilities distinctly required for each task. In contrast, the RTs of Syn and Spe were highly correlated (*r* = 0.82, *P* < 0.0001; **Figure 1D**). Indeed, the RTs of Syn and Spe were positively correlated in all three of the groups (*r* > 0.7, *P* < 0.005). These results indicate that the RTs of each participant were related to general cognitive processes common to both tasks. Moreover, the correlation coefficient between RTs of Syn and Spe was significantly larger compared to that between the accuracy of Syn and Spe (*Z* = 4.4, *P* < 0.005), confirming the differential natures of these two behavioral measurements. These results suggest that a participant’s *accuracy* of each task reflected individual abilities employed for each task. On the other hand, RTs were metrics that reflected more general cognitive abilities, such as reading proficiency. Once a structural property which showed any group difference related to L2 acquisition was found, we further examined its correlation with the accuracy of Syn or Spe to reveal which ability was dominantly reflected. Moreover, we also examined its correlation with the RTs to reveal if this structural property was related to general cognitive abilities.

We also examined the verbal fluency data in L1, and found a consistent trend among groups with the Syn and Spe performances. Behavioral data of the verbal fluency task are shown in **Table 1**. A one-way ANOVA showed a significant effect of group [*F*(2,44) = 4.0, *P* < 0.05]. The Senior (High) group produced significantly larger numbers of words than the Junior (High) group [*t*(23) = 2.8, *P* < 0.005, *d* = 1.1, 1 – β = 0.82], surviving Bonferroni correction for the three comparison pairs (significance level at *α* = 0.017); the Senior (High) group also produced larger numbers of words than the Senior (Low) group [*t*(31) = 2.0, *P* = 0.03, *d* = 0.7, 1 – β = 0.52]. For the performance-matched groups, i.e., the Junior (High) group and the Senior (Low) group, there was no significant difference [*t*(30) = 0.8, *P* = 0.2]. To examine how syntactic abilities in L2 were related to overall proficiency in L1, we further examined the correlations between the verbal fluency in L1 and the accuracy of Syn for each group. While correlations were not significant for the Senior (High) (*r* = 0.23, *P* = 0.4) and Senior (Low) (*r* = 0.10, *P* = 0.7) groups, a significant positive correlation was present for the Junior (High) group (*r* = 0.68, *P* < 0.01). These results suggest that syntactic abilities in L2 may depend on L1 proficiency in early acquisition stages (DOE ≈ 1.5 years), but this relationship became weaker in later acquisition stages (DOE ≈ 4.5 years).

### Group Differences along the Tract of the Arcuate

**FIGURE 2:**
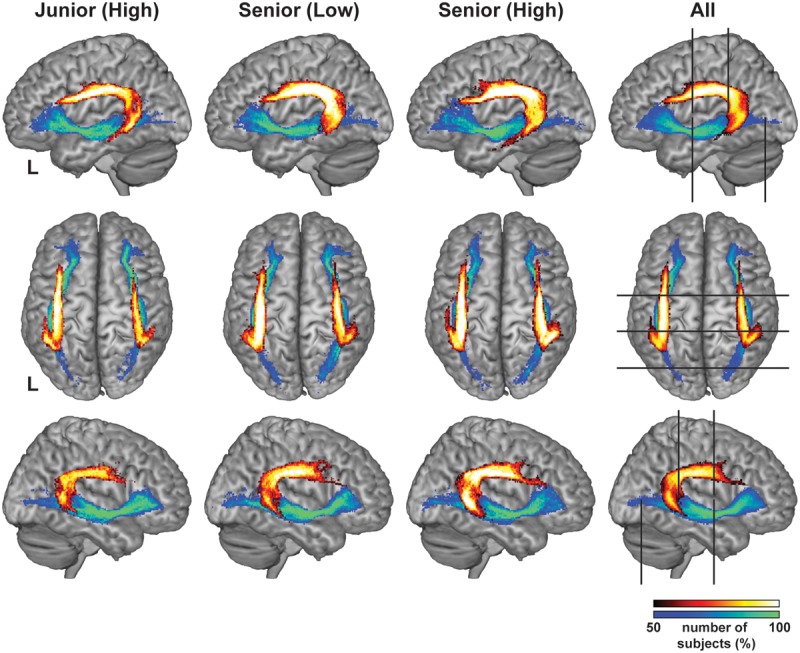
**The Arcuate and IFOF reconstructed with diffusion MRI.** The probability maps of population for the Arcuate and IFOF in the MNI space. The pathways were thresholded to show the results of the tracking present in more than half of the participants in each group. The color scales denote the number of participants (%): black–red to yellow–white for the Arcuate, and blue to green for the IFOF. Note that the Arcuate in the Senior (High) and Senior (Low) groups was clearly thicker than that in the Junior (High) group, which was evident from both left lateral (top row) and top (middle row) views. Similar tendency was observed in the right hemisphere from the right lateral (bottom row) view. To examine the overall profile while excluding highly variable regions among individuals, we first set candidate regions for selecting regions of interest (ROIs): a region of 35 mm for the Arcuate (*y* = –40 ∼ –6), and a region of 70 mm for the IFOF (*y* = –75 ∼ –6). The right-most panel shows the candidate regions, which are bounded by the pairs of black lines. We further selected ROIs at the portion with the most uniform thickness in each pathway to quantify the Arcuate and IFOF (see the “Materials and Methods” section), as shown in **Figure 3**.

The Arcuate and IFOF were successfully tracked in both hemispheres for every participant, and the tracking was basically similar among groups. In all groups, the Arcuate connected the frontal and temporal regions with a similar curvature, and the IFOF connected the frontal and occipital/temporal regions through a narrower portion near the external capsule (**Figure 2**). While these overall characteristics were the same for the left and right hemispheres, the left Arcuate was thicker than the right Arcuate. From both lateral and top views, the Arcuate was consistently thicker and extended more anteriorly in the left hemisphere. The Arcuate of the Senior (High) and Senior (Low) groups was thicker than that of the Junior (High) group, which was evident from both views. Such group differences were not observed for the IFOF in either hemisphere.

**FIGURE 3:**
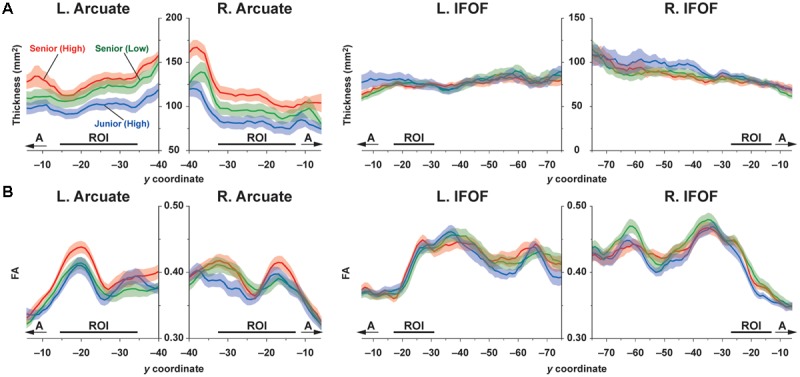
**Profiles of the thickness/FA of the dorsal and ventral pathways in each group. (A)** The profiles of thickness for the Arcuate and IFOF. **(B)** The profiles of FA for the Arcuate and IFOF. The thickness and FA were averaged among the participants in each of the Junior (High), Senior (Low), and Senior (High) groups, as shown in blue, green, and red, respectively. Data are shown for the range of *y* = –40 ∼ –6 for the Arcuate and *y* = –75 ∼ –6 for the IFOF (bounded by the pairs of black lines in **Figure 2**) in the MNI space. Note that the thickness of the Arcuate in the Senior (High) group is larger than that in the Junior (High) group in both hemispheres. Moreover, FA in the *left* Arcuate in the Senior (High) group is higher than those in the other two groups. No clear group difference was found for the IFOF in either hemisphere. The SEMs in each group are shown as shaded bands in each color. The positions of ROIs are indicated by the black lines above the axes. A, anterior.

To examine the overall profile while excluding highly variable regions among individuals, we first set the candidate regions for ROI selection: the region of 35 mm for the Arcuate (*y* = -40 ∼-6), and the region of 70 mm for the IFOF (*y* = -75 ∼ -6) (**Figure 2**). As regards the Arcuate, the candidate region was selected where the pathway was relatively straight, excluding the curved or branching portions. The candidate region for the IFOF was also selected where the pathway was straight, excluding the narrower portion near the external capsule, resulting in a longer candidate region than for the Arcuate. The thickness of the Arcuate in the Senior (High) and Senior (Low) groups was significantly larger than that of the Junior (High) group throughout the candidate region in both hemispheres, as indicated by the non-overlapping error bars (mean ± SEM) (**Figure 3A**). In contrast, for the thickness of the IFOF, no clear group difference was observed in either hemisphere. For most of the candidate regions in the Arcuate and IFOF, the thickness was basically uniform in both hemispheres. We selected one-dimensional ROIs, where the thickness was most uniform (see the “Materials and Methods” section). Because the thickness was independent of FA, our ROIs were free from sampling bias of extracting regions with particularly higher or lower FA. We also plotted FA in the same regions in each hemisphere, and found that FA of the left Arcuate in the Senior (High) group was higher than those of the other two groups, especially in the anterior regions, as indicated by the non-overlapping error bars (**Figure 3B**). In contrast, FA of the right Arcuate did not show such clear group differences. In regard to the IFOF, however, no clear group difference was observed. In these ROIs, FA in the Arcuate showed some modulations, while FA in the IFOF showed relatively sharp antero-posterior changes. Nevertheless, the overall tendency of thickness or FA was similar among the three groups throughout the candidate regions in both hemispheres.

### Distinct Group Differences in the Structural Properties of the Left Arcuate

**FIGURE 4:**
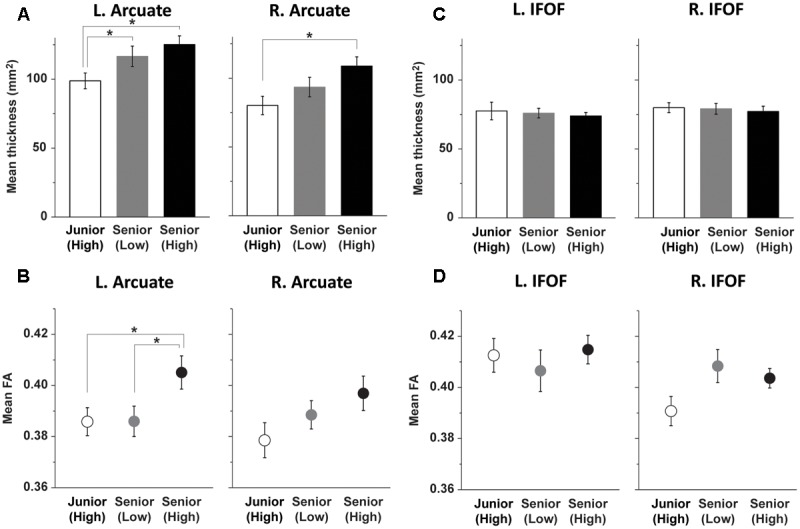
**Group differences in the structural properties of the left Arcuate. (A)** Age-related group differences on the thickness of the left dorsal pathway. **(B)** Performance-related group differences on FA in the left dorsal pathway. **(C,D)** The ventral pathway without group differences. The Junior (High), Senior (Low), and Senior (High) groups are indicated by bars/dots with white, gray, and black shades, respectively. Error bars indicate the SEM for the participants, and asterisks denote statistical differences at *P* < 0.05 (*post hoc t*-test after ANOVA).

For all the participants, the ROIs were placed at the same position of each pathway in the MNI space. Within these ROIs, we calculated the mean thickness of the Arcuate, and confirmed the leftward laterality (**Figure 2**). A two-way rANOVA [group × hemisphere (left, right)] indicated significant main effects of group [*F*(2,44) = 4.9, *P* = 0.01] and hemisphere [*F*(1,44) = 27, *P* < 0.001], without an interaction [*F*(2,44) = 0.3, *P* = 0.7] (**Figure 4A**). Considering the potential relationships between the thickness in the left and right hemispheres of the same participants, we used a linear mixed-effects model analysis [fixed effects: group, hemisphere; random effects: subject], confirming significant effects of group [χ^2^(2) = 9.8, *P* = 0.007] and hemisphere [χ^2^(1) = 28, *P* < 0.0001]. Indeed, in all of the three groups, the mean thickness in the ROI of the Arcuate was significantly larger in the left than in the right hemisphere (*P* ≤ 0.02) (one-sided *t*-tests). As regards the group differences, the mean thickness of the left Arcuate for the Senior (High) group was significantly larger than that of the Junior (High) group [*t*(27) = 3.1, *P* = 0.002, *d* = 1.2, 1 – β = 0.94] (one-sided *t*-tests); the mean thickness for the Senior (Low) group was also larger than that of the Junior (High) group [*t*(30) = 1.8, *P* = 0.04, *d* = 0.7, 1 – β = 0.59]. There was no significant difference between the Senior (High) and Senior (Low) groups [*t*(31) = 0.86, *P* = 0.2]. Regarding the mean thickness of the right Arcuate, the thickness for the Senior (High) group was significantly larger than that for the Junior (High) group [*t*(27) = 3.0, *P* = 0.003, *d* = 1.2, 1 – β = 0.93]. There was no significant difference between the Senior (Low) and Junior (High) groups [*t*(30) = 1.3, *P* = 0.10] or between the Senior (High) and Senior (Low) groups [*t*(31) = 1.6, *P* = 0.06]. No significant effect of gender was observed in either hemisphere, according to one-way ANOVAs [*F*(1,45) < 0.4, *P* > 0.5].

Based on the results of FA shown in **Figure 3B**, we focused on group differences in the left Arcuate. For all voxels (with FA values of 0.2 or higher) within the tract at the ROI of the left Arcuate, we calculated the mean FA, which was clearly higher in the Senior (High) group than in the other two groups (**Figure 4B**). A one-way ANOVA for FA in the left Arcuate indicated a significant main effect of group [*F*(2,44) = 3.3, *P* < 0.05]. More specifically, FA in the left Arcuate for the Senior (High) group was significantly higher than that for the Junior (High) group [*t*(27) = 2.2, *P* = 0.016, *d* = 0.9, 1 – β = 0.75]; FA in the left Arcuate for the Senior (High) group was also higher than that for the Senior (Low) group [*t*(31) = 2.2, *P* = 0.019, *d* = 0.8, 1 – β = 0.72]. There was no significant difference between the Senior (Low) and Junior (High) groups [*t*(30) = 0.02, *P* = 0.5]. Regarding FA in the ROI of the right Arcuate, a one-way ANOVA did not show a significant main effect of group [*F*(2,44) = 2.0, *P* = 0.15]. No significant effect of gender was observed for FA in either hemisphere, according to one-way ANOVAs [*F*(1,45) < 0.2, *P* > 0.7].

In regard to the IFOF, neither the mean thickness nor mean FA in the ROI showed a group difference (**Figures 4C,D**). A one-way ANOVA for the thickness did not show a significant main effect of group in either hemisphere [*F*(2,44) < 1.7, *P* > 0.8]. In addition, a one-way ANOVA for FA in the IFOF did not show a significant main effect of group in either hemisphere [*F*(2,44) < 2.6, *P* > 0.05]. Major structural properties, i.e., the thickness and FA, in the ROI of IFOF in both hemispheres were similar among the three groups.

### FA in the Left Arcuate Was Selectively Correlated with the Accuracy of Syn

**FIGURE 5:**
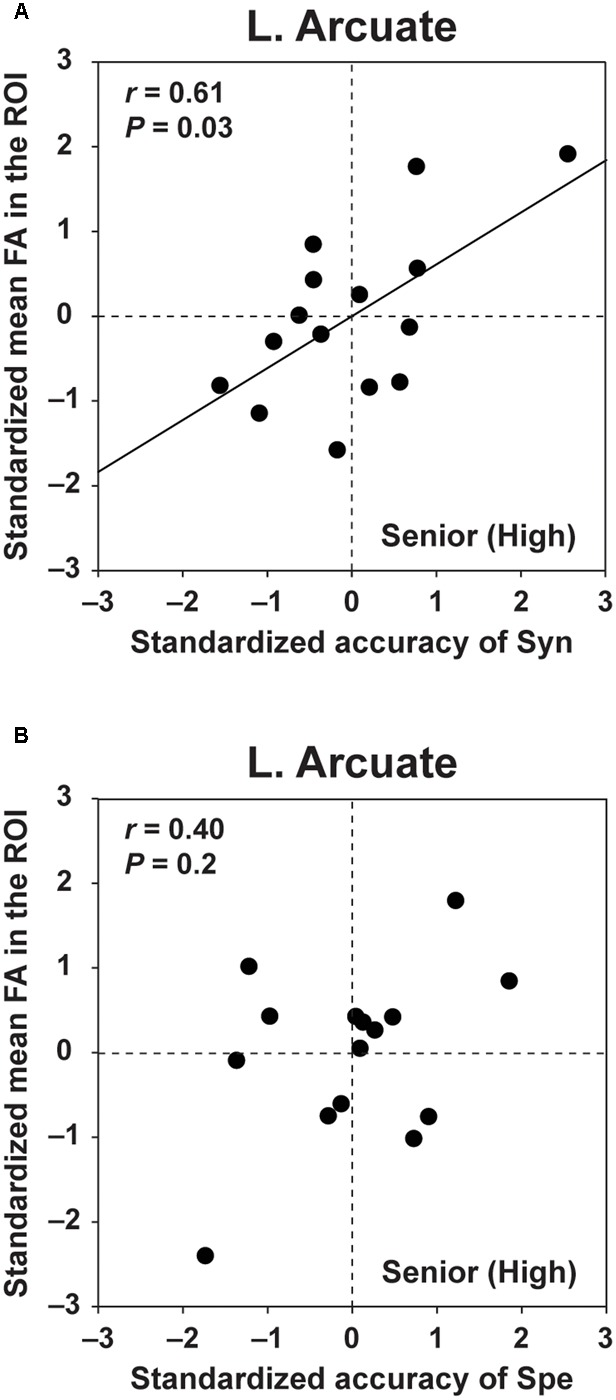
**FA in the left Arcuate was selectively correlated with the accuracy of Syn. (A)** The scatter plots of the standardized accuracy of Syn and the standardized mean FA in the left Arcuate. The ROIs in the left Arcuate were determined for each participant in the Senior (High) group. The effects of the standardized accuracy of Spe, gender, and laterality quotient (LQ) were removed. **(B)** The scatter plots of the standardized accuracy of Spe and the standardized mean FA in the left Arcuate. The effects of the standardized accuracy of Syn, gender, and LQ were removed. FA in the left Arcuate was correlated with the accuracy of Syn, but not with the accuracy of Spe.

For FA in the left Arcuate, which showed group differences between the Senior (High) and the other two groups (see **Figure 4B**), we examined what aspect of L2 abilities was actually related to FA. We performed the following analyses for the Senior (High) group, whose task performances were higher and thus most reliable for dissociating the linguistic abilities required by Syn or Spe. ROIs were selected for each participant as described in the “Materials and Methods” section. We performed partial correlation analyses between the standardized accuracy of Syn and the standardized FA in the left Arcuate, removing the effects of the accuracy of Spe, LQ, and gender. Regarding the accuracy of Syn, we found a significant correlation with FA in the left Arcuate (*r* = 0.61, *P* = 0.03) (**Figure 5A**). In contrast, the accuracy of Spe was not significantly correlated with FA in the left Arcuate (*r* = 0.40, *P* = 0.2), according to the partial correlation analysis removing the effects of the accuracy of Syn, LQ, and gender (**Figure 5B**). In addition, no significant correlation was found between FA in the left Arcuate and verbal fluency in L1 (*r* = 0.24, *P* = 0.4) in the partial correlation analysis removing the effects of LQ and gender. Moreover, no significant correlation was found between FA in the left Arcuate and the RTs of Syn (*r* = 0.17, *P* = 0.6), according to a partial correlation analysis removing the effects of the RTs of Spe, LQ, and gender. These results indicate that increased FA in the left Arcuate for the Senior (High) group was related mainly to the enhanced syntactic abilities in L2, irrespective of L1 performances or other general measures examined here.

## Discussion

In the three groups of high school students, we obtained the following results. First, performance-related group differences were found only for FA in the left Arcuate. More specifically, the Senior (High) group, who had higher L2 abilities, showed higher FA in the left Arcuate than the Senior (Low) and Junior (High) groups (**Figures 3B, 4B**). Moreover, the mean FA in the ROI of the left Arcuate of the Senior (High) group was significantly correlated with the accuracy of Syn (**Figure 5A**), but not with the accuracy of Spe (**Figure 5B**) or with verbal fluency in L1, indicating that increased FA in the left Arcuate was related mainly to the enhanced syntactic abilities in L2. Secondly, age-related group differences were found for the thickness of the left Arcuate. The thickness for the Senior (High) and Senior (Low) groups was larger than that for the Junior (High) group (**Figures 3A, 4A**), indicating that the left Arcuate was still developing in adolescence. Thirdly, these differential performance-related and age-related signatures were evident on the left Arcuate alone, in contrast to the right Arcuate that showed only mild differences in thickness (**Figures 3A, 4A**), and to the bilateral IFOF that lacked either signature (**Figures 3, 4C,D**). To the best of our knowledge, our study is the first to report that plasticity during the adolescent years was markedly different between the dorsal and ventral language-related pathways, the former of which was related to syntactic abilities. In summary, we showed that the left dorsal pathway, which has been reported to be more immature than the ventral pathway from early infancy, continued to grow thicker with increasing age at least until the adolescent years. Further, by showing the group difference between the Senior (High) and Senior (Low) groups, whose DOEs were the same, we revealed that performance differences were reflected in the FA in the left dorsal pathway, independent of age and the duration of experience. These results indicate that the left dorsal pathway is a major neural network supporting syntactic abilities.

Within the same part of the left dorsal pathway, we observed the dissociated performance-related and age-related group differences on FA and the thickness, respectively. These results provide new insights into the plasticity of these two distinct structural properties in the left Arcuate. FA and the volume of the Arcuate have been reported to change during the adolescent years (Lebel and Beaulieu, 2011), but it has remained unclear whether or not these properties change in accordance with the development of specific abilities. Our present results suggest that not age or maturation *per se* but the gradual improvement of performances in syntactic abilities during adolescence was related to FA. The increased FA in accordance with certain learning or L2 exposure has been reported also in other pathways. For instance, an increase in FA in the genu of the corpus callosum was correlated with the overall L2 performances after a 9-month intensive course of spoken and written Chinese (Schlegel et al., 2012). Moreover, an increase in FA in the white matter underlying the right IFG was correlated with vocabulary test scores after a 16-week period of vocabulary training in English (Hosoda et al., 2013). In our previous study, focusing on students with better orthographic knowledge (80% or higher accuracy in the Spe task), we showed that individual differences in the accuracy of Syn were reflected in FA in the left Arcuate (Yamamoto and Sakai, 2016). In the present study, analyzing students with a wider range of L2 abilities and ages, we demonstrated that FA in the left Arcuate was higher in students with higher L2 abilities than in students with lower abilities at the same age, whose FA was not significantly different from performance-matched younger students. Further, we showed that FA in the left Arcuate of the Senior (High) group was positively correlated with the accuracy of the syntactic task, but not with the accuracy of the spelling task. These results indicate that the higher FA in the left dorsal pathway was mainly related to the higher syntactic abilities in L2. As described in the “Materials and Methods” section, the Syn task was designed to test argument structures and related syntactic knowledge in English, and cannot be correctly answered by semantical/pragmatic cues, or by internally translating English sentences into Japanese. Indeed, the accuracy of Syn and L1 verbal fluency was not significantly correlated in the Senior (High) and Senior (Low) groups. Future studies with tasks that can accurately assess individual syntactic abilities in L1 will be needed to reveal how L1 syntactic abilities, as well as neural networks supporting these abilities, facilitate (or hamper) the acquisition of a new language. Moreover, it would be also important to elucidate how the development in other aspects of linguistic abilities is related to the plasticity in the left dorsal pathway.

Another important issue is whether non-cognitive abilities are related to the development of the language-related structures. A previous study on L2 learning in infants indicated the impact of social interaction, by showing that infants who received “live-person” sessions on Mandarin performed significantly better on Mandarin phonetic perception tests, compared to infants who received the identical information via television or audiotape but showed no learning effects (Kuhl et al., 2003). These social factors, as well as other non-cognitive factors such as motivation and self-confidence, may have impacts on L2 acquisition in adolescent students and adults as well (Dörnyei, 2003; Gardner, 2010). One of the directions of future studies would be to investigate how such individual traits affect one’s acquisition experience, which may further modify functional and structural networks. It would also be important to dissociate the effects of non-cognitive abilities on language-related networks from those of cognitive abilities on the left dorsal pathway, whose critical involvement in linguistic information processing has been shown by previous studies (Ohta et al., 2013; Yamamoto and Sakai, 2016), and confirmed in the present study.

Here, we showed that the thickness of the left Arcuate was larger in the Senior (High) and Senior (Low) groups than in the Junior (High) group, indicating age-related group differences. This macro-structural property of the left Arcuate had plasticity associated with age, which may reflect biological maturation, as well as common and specific experiences that students underwent during the ages of 13–17. A recent longitudinal study reported that children’s structural connectivity, which was obtained before they learned to read (i.e., before age 5), predicted the spatial profile of functional activations in the Visual Word Form Area (VWFA) after they learned to read (i.e., after age 8), suggesting that connectivity precedes the functional development (Saygin et al., 2016). These results raise an interesting hypothesis that experience interacts with the micro-structural development of a pathway whose structural connectivity has already developed. A more detailed picture of the developmental mechanism of language-related structures would be provided by closely linking the development of linguistic abilities and that of white matter pathways, as well as the structures of connected cortical regions, which can be examined by such methods as VBM and the myelin mapping technique (Glasser and Van Essen, 2011). Our present study showed both age-related and performance-related group differences in adolescent participants, suggesting the importance of future longitudinal studies with various structural and functional measurements.

In addition to the fronto-temporal segment examined here, the arcuate fasciculus is also composed of the fronto-parietal and temporo-parietal segments (Catani et al., 2005). Thiebaut de Schotten et al. (2014) have suggested that the temporo-parietal segment has plasticity associated with learning even in adults, based on their finding that its FA was higher in a group of ex-illiterates, who lacked access to schools during childhood for social reasons and learned to read during adulthood, in contrast to a group of illiterates, who never learned to read. They also showed that FA was correlated with activations in the two regions, i.e., the left VWFA and planum temporale, connected to the angular/supramarginal gyri by this segment. As the authors of this study discussed in their subsequent paper (Dehaene et al., 2015), these results might have been influenced by multiple factors, including the motivations, self-confidence, socioeconomic status, and professions of the participants. Indeed, differences in FA between ex-illiterates and illiterates might be present before ex-illiterates voluntarily begin to learn reading. Future studies should attempt to verify whether or not “learning to read improves the structure” or not. To examine such causal changes in the brain, longitudinal studies that track neural indices and behavioral measures, together with multiple regression analyses that de-correlate confounding variables, would be critical (Galván, 2010; Dehaene et al., 2015). Note that our present study does not intend to suggest causal influences of L2 acquisition on structural properties. Rather, we demonstrated here that the performance-related group differences, which were separated from the duration of L2 experience (i.e., DOE) as well as from the age-related group differences, were predicted by FA in the left dorsal pathway.

## Author Contributions

All authors listed have made substantial, direct and intellectual contribution to the work, and approved it for publication.

## Conflict of Interest Statement

The authors declare that the research was conducted in the absence of any commercial or financial relationships that could be construed as a potential conflict of interest.

## Footnotes

1 https://www.r-project.org/

2 http://people.cas.sc.edu/rorden/mricron/index.html

3 https://cran.r-project.org/web/packages/cocor/

4 https://cran.r-project.org/web/packages/ppcor/

5 https://cran.r-project.org/web/packages/pwr/

6 https://cran.r-project.org/web/packages/lme4/

7 https://cran.r-project.org/web/packages/car/
